# An observational study of the use of beclomethasone dipropionate suppositories in the treatment of lower urinary tract inflammation in men

**DOI:** 10.1186/s12894-016-0144-8

**Published:** 2016-06-08

**Authors:** Giorgio Bozzini, Marco Provenzano, Nicolò Buffi, Mauro Seveso, Giovanni Lughezzani, Giorgio Guazzoni, Alberto Mandressi, Gianluigi Taverna

**Affiliations:** Departmentt of Urology, Humanitas Mater Domini, Via Gerenzano 2, I - 21053 Castellanza, Varese Italy; Humanitas University, Milan, Italy; Department of Urology, Humanitas Research Hospital, Milan, Italy

**Keywords:** Nonbacterial prostatitis, Beclomethasone dipropionate, Lower urinary tract inflammation

## Abstract

**Background:**

Nonbacterial prostatitis, together with chronic pelvic pain syndrome, accounts for 90–95 % of prostatitis cases. Anti-inflammatory medications are commonly used to reduce storage/inflammatory symptoms that can deteriorate quality of life. The purpose of this study was to observe the efficacy and safety of beclomethasone dipropionate rectal suppositories (Topster®) in inflammations of the lower urinary tract in men.

**Methods:**

Patients underwent diagnostic and therapeutic protocols according to current evidence-based practice. Efficacy assessments: voiding parameters, perineal pain, International Prostate Symptom Score (IPSS), digital rectal examination (DRE). Adverse events and patient compliance were recorded throughout the study.

**Results:**

One hundred eighty patients were enrolled, mean age 52 ± 14.97. Most frequent diagnosis: nonbacterial prostatitis (85 %). All patients completed visits 1 and 2. All patients were treated with beclomethasone dipropionate (BDP) suppositories, 136/180 also with *Serenoa repens* (SR) extract. Antibiotics were rarely required.

162/180 patients presented clinically significant improvements and terminated treatment.

Mean change vs. baseline in voiding frequency: −3.55 ± 2.70 n/day in patients taking only BDP and −3.68 ± 2.81 n/day in those taking both BDP and SR (*P*<.0001 in both groups). Uroflowmetry improved significantly; change from baseline 3.26 ± 5.35 ml/s in BDP only group and 5.61 ± 7.32 ml/s in BDP + SR group (*P* = 0.0002 for BDP, *P*<.0001 for BDP + SR). Urine stream normal in 35 % of patients at visit 1 and 57.22 % of patients at visit 2. Mean change in perineal pain, on 0–10 VAS, −0.66 ± 2.24 for BDP only group (*P* = 0.0699) and −1.37 ± 2.40 for BDP + SR group (*P*<.0001). IPSS increased at visit 2. No adverse events were reported.

For all parameters, none of the comparisons between groups was found to be statistically significant.

**Conclusion:**

This study confirmed the drug’s good safety profile. We also observed an improvement in the main storage symptoms and clinical findings associated with lower urinary tract inflammation in patients treated with beclomethasone dipropionate suppositories.

## Background

Inflammation of the lower urinary tract, especially of the prostate, commonly affects men of a wide age range, with detrimental repercussions on quality of life [1–3]. Symptoms include pelvic pain and a variable degree of voiding and sexual dysfunction [4, 5].

Although prostatitis is historically considered mainly a bacterial disease, its most common form is chronic prostatitis/chronic pelvic pain syndrome, accounting for 90–95 % of prostatitis cases [1, 2, 6].

Traditional medical therapy for prostatitis is centered on treating infection with antimicrobials, although less than 10 % of prostatitis cases are bacterial, and on alleviating symptoms with NSAIDS, alpha-blockers, 5-Alpha-Reductase Inhibitors, and phytotherapy [2, 6, 7].

Currently, corticosteroids are not considered “standard of care” for treating prostatitis. However, in a double-blinded, randomized, parallel study, 160 patients presenting with chronic nonbacterial prostatitis received prednisone and levofloxacin or levofloxacin and placebo; significant differences between the two groups as well as between pre- and post-treatment (*P* < 0.01) were found for total NIH-CPSI score, pain index, voiding index and quality of life [5]. Currently beclomethasone dipropionate suppositories is a part of the standard practice in the hospital.

Furthermore, new formulations of corticosteroids have been developed to limit systemic activity and reduce corticosteroid adverse events [8–12]. Second-generation oral or rectal corticosteroids such as beclomethasone dipropionate have high topical anti-inflammatory efficacy in the gut and minimal systemic bioavailability due to low absorption and highly efficient first-pass hepatic inactivation [10, 11]. A systematic review of rectal therapies for distal forms of ulcerative colitis found that a greater percentage of patients receiving 5-aminosalicylic acid or corticosteroid rectal formulations obtained therapeutic benefit after treatment compared with placebo [13]. The overall safety profile of rectal therapies was favorable and treatment with beclomethasone dipropionate did not increase the incidence of steroid-related adverse events [9, 10, 12, 13].

The objectives of this study were to collect safety data and observe the effects of beclomethasone dipropionate (Topster®, SOFAR S.p.A., Milan, Italy) rectal suppositories on symptoms associated with lower urinary tract inflammation.

## Methods

This was a prospective, observational, single-center study performed on outpatients referred to a high-volume academic teaching hospital in Italy. The study was approved by the local Ethics Committee and written informed consent was obtained from all patients.

Male patients presenting with storage/inflammatory symptoms of the lower urinary tract (pelvic pain, voiding and sexual dysfunction) were observed as they underwent diagnostic and therapeutic protocols according to clinical practice. Subjects affected by coagulation impairments, cardiovascular or pulmonary comorbidities were excluded from the observation, along with those who had undergone a prostatic biopsy within the previous 14 days.

Semen and urine cultures were performed at baseline to determine if the inflammation was triggered by an infection, and whether an antibiotic was therefore indicated.

The following parameters were assessed at each visit: voiding frequency, uroflowmetry, urine stream, perineal pain, prostate-specific antigen (PSA), International Prostate Symptom Score (IPSS) [14] and digital rectal examination (DRE) (evaluation of prostate size, temperature and consistency).

At visit 1, baseline clinical assessments were performed and therapy was prescribed according to current evidence-based practice [1, 2, 15, 16]. Patients were then re-evaluated at visit 2, after the end of the treatment course.

A patient was considered “responder” to therapy following a clinically significant improvement of the evaluated parameters (i.e. voiding frequency, uroflowmetry, urine stream, perineal pain) and the patient’s impression of a good clinical outcome.

### Drugs prescribed and rationale

Beclomethasone dipropionate (BDP) rectal suppositories (3 mg, 1 supp. once a day) were prescribed for the relief of inflammation-related storage symptoms. The duration of treatment varied depending on the patient’s conditions: 10-day courses were prescribed to patients with mild symptoms (e.g. perineal pain on a 0–10 VAS between 4 and 6, voiding frequency less than 10 times a day) or to those expected not to comply with longer treatment; 20-day courses in other cases (severe symptoms, high compliance expected).

Serenoa repens (SR) 320 mg (1 tab. a day for 60 days) was suggested as adjuvant treatment for voiding and storage symptoms due to prostatic hypertrophy and inflammation. We used the only formulation registered as a drug (and not as a dietary supplement) in Italy (Permixon 320 mg®), as requested by the Ethics committee due to the current Literature evidence. It was not prescribed in patients who had already undergone this treatment with unsatisfactory outcomes.

An antibiotic course was prescribed in case of bacterial infection (positive seminal fluid and/or urine cultures). The specific antibiotic was recommended according to antibiogram results, patient preference regarding route of administration and current guidelines for the outpatient treatment of lower urinary tract infections [15, 16].

A diet (no alcohol, beer or spicy food) and/or hygiene rules (e.g. avoid cycling/riding, prolonged/interrupted sexual intercourse, constipation/diarrhea) were also recommended.

Safety monitoring consisted in gathering all adverse reactions occurring during the study.

All statistical tables, figures, listings and analyses were produced using SAS® for Windows release 9.4 (64-bit) or later (SAS Institute Inc., Cary, NC, USA). Box plots for IPSS score, uroflowmetry, urination frequency, PSA and perineal pain were produced by visit and by type of therapy (BDP suppositories only for patients in group A, or BDP suppositories plus SR for patients in group B). Differences between visit 2 and baseline were analyzed by means of a paired t-test in case of normal data distribution, or a non-parametric Wilcoxon signed rank sum test otherwise. A two independent samples t-test was performed in order to compare the two treatment groups if the changes vs. baseline were normally distributed. Otherwise, the analogous non-parametric test (Wilcoxon-Mann–Whitney test) was used.

## Results

One hundred eighty patients were enrolled in this study between January and December 2013 and all of them completed both visit 1 and visit 2. One hundred thirty-six patients were treated with both BDP and SR (Group B), whereas 44 were treated with BDP only (Group A).

Patients averaged 52 years of age (SD 14.9, range 22–87) and nonbacterial prostatitis was by far the most frequent diagnosis, affecting 89.7 % of patients treated with BDP + SR (Group B) and 70.4 % of those treated with BDP (Group A) (Table 1). The mean duration of symptoms was 2.7 ± 1.8 months (range 1–12 months) and the number of previous episodes 1.2 ± 1.2 (range 0–6). Approximately 26 % of patients had undergone previous treatment with antibiotics, of which ciprofloxacin, cefixime and levofloxacin were the most commonly prescribed. Urine and semen cultures at visit 1 were positive in 13 (7.2 %) and 12 (6.6 %) patients respectively.

**Table 1:** Diagnosis

		Total		Treatment			
				Group A		Group B	
		(*N* = 181)		(*N* = 45)		(*N* = 136)	
		*N*	%	*N*	%	*N*	%
Diagnosis at Baseline	Chlamydial Urethritis	2	1.10	1	2.27	1	0.74
	Chronic Nonbacterial Prostatitis	3	1.66	0	0.00	3	2.21
	Nonbacterial Prostatitis	154	85.08	31	70.45	122	89.71
	Nonbacterial Prostatitis (First episode)	3	1.66	1	2.27	2	1.47
	Post Endoscopic Resection Urethritis	11	6.08	7	15.91	4	2.94
	Results of pyelonephritis	1	0.55	0	0.00	1	0.74
	Urethritis	7	3.87	4	9.09	3	2.21

All 180 patients underwent at least one course of therapy with BDP suppositories. Serenoa repens 320 mg (saw palmetto extract) was prescribed to 136 patients. Antibiotics were prescribed to only one patient (Table 2). The other patients with positive urine or semen cultures were already taking the proper antibiotic prescribed by their general practioner.

**Table 2:** Prescribed medications

			Total		Treatment			
					Group A		Group B	
			(*N* = 181)		(*N* = 45)		(*N* = 136)	
			*N*	%	*N*	%	*N*	%
Visit	Drug	Dosage	181	-	45	-	136	-
Visit 1	Patients visited -	-						
	Doxycycline 400	1 tab for 7 days	1	0.55	1	2.27	0	0.00
	Serenoa repens 320 mg	1 tab for 40 days	30	16.67	0	0.00	30	22.06
		1 tab for 60 days	106	58.89	0	0.00	106	77.94
	Beclomethasone dipropionate suppositories	1 supp. for 10 days	28	15.56	1	2.27	27	19.85
		1 supp. for 20 days	152	84.44	43	97.73	109	80.15

At visit 2, all patients reported being compliant with the prescribed therapies and suggestions concerning diet and lifestyle.

Mean time elapsed between visits was 99.6 ± 38.3 days (range 27–179 days).

### Efficacy results

One hundred sixty-two of the 180 patients treated with BDP presented clinically significant improvements and terminated treatment. Further therapeutic interventions were required for only 18 patients.

**Fig. 1:**
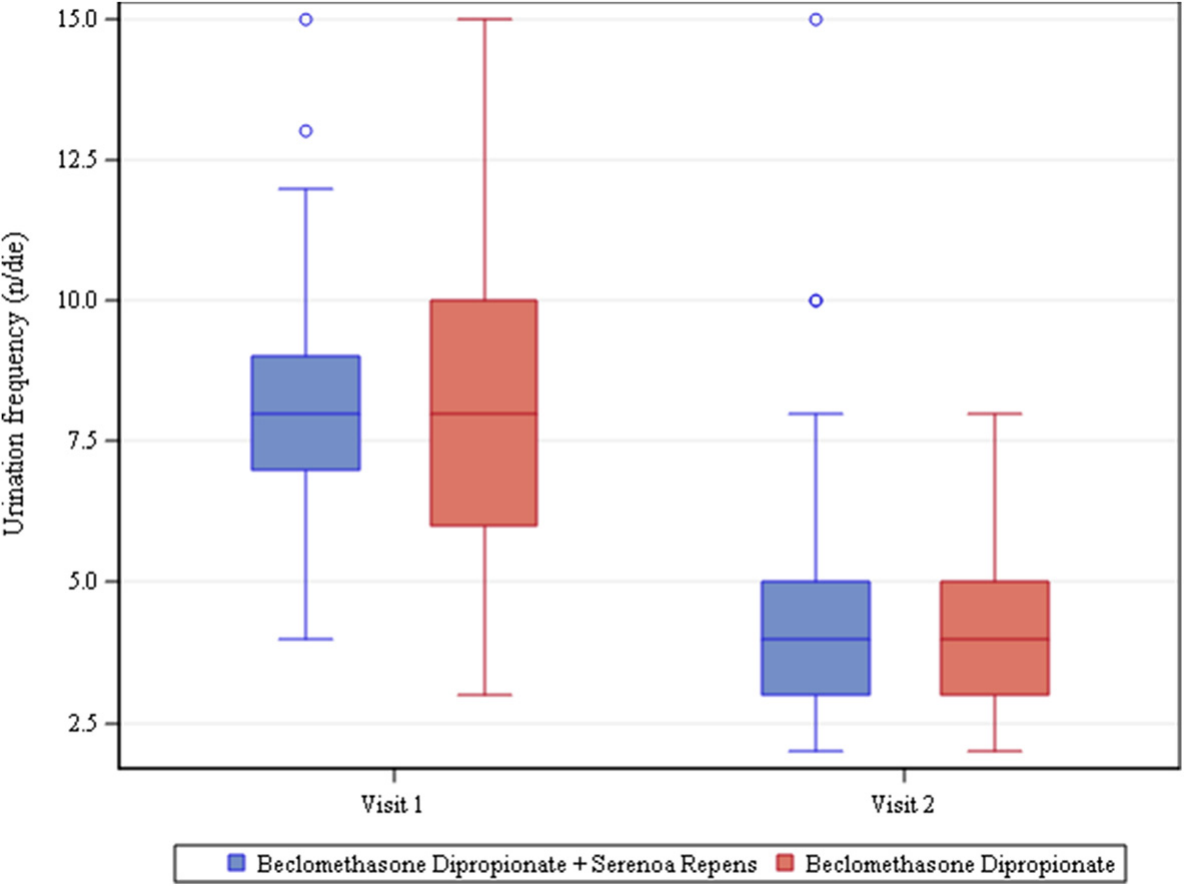
Voiding frequency. The bottom of each box is the 25th percentile (Q1), the top is the 75th percentile (Q3), and the internal line is the median. The whiskers indicate variability outside the upper and lower quartiles, i.e. scores outside the middle 50 %. A circle outside of this range is an outlier, an observation that is distant from others

The study evidenced noteworthy improvements in voiding parameters. Considering voiding frequency, the changes from baseline were found to be statistically significant (*P*<.0001) in both groups (−3.5 ± 2.7 n/day in patients taking only BDP suppositories and −3.6 ± 2.8 n/day in those taking both BDP and SR), whereas the difference in mean change between the two groups was not (p-value = 0.8560) (Fig. 1).

**Fig. 2:**
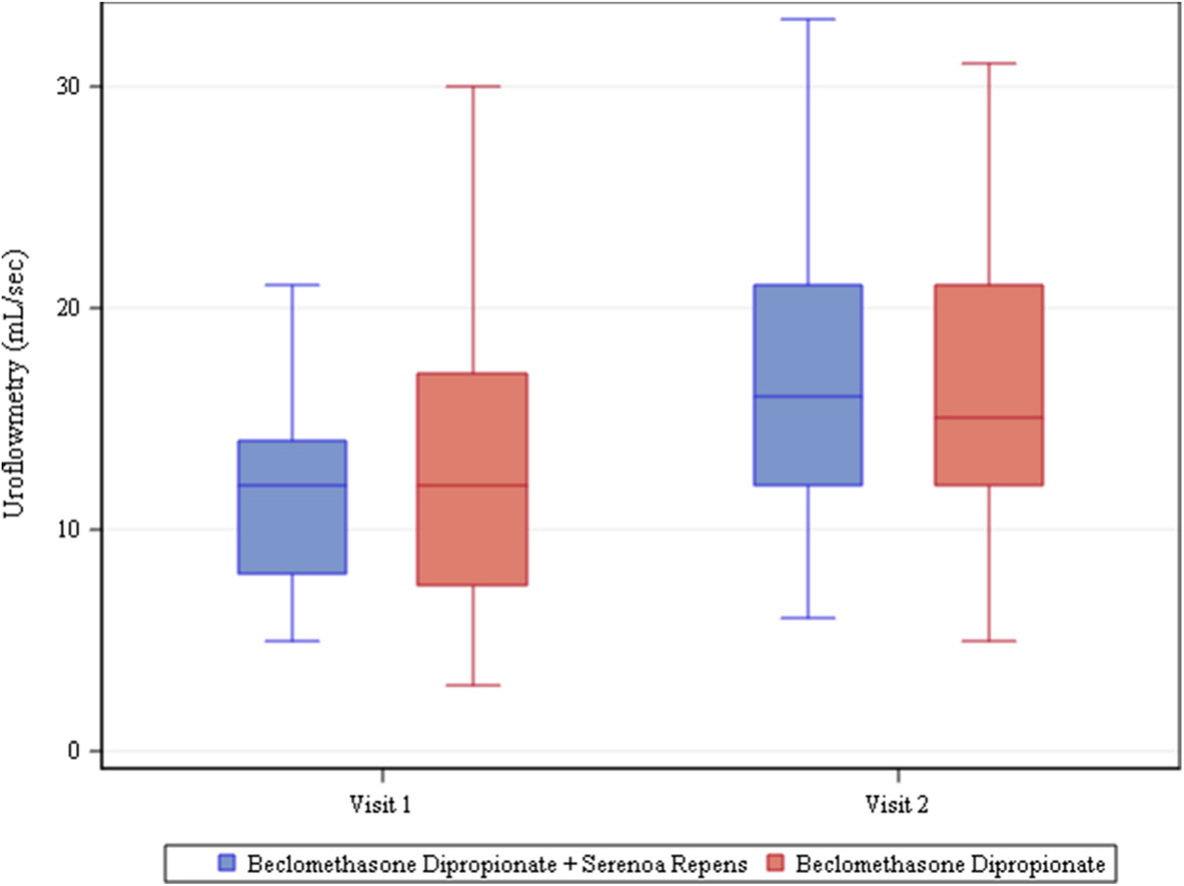
Uroflowmetry. The bottom of each box is the 25th percentile (Q1), the top is the 75th percentile (Q3), and the internal line is the median. The whiskers indicate variability outside the upper and lower quartiles, i.e. scores outside the middle 50 %. A circle outside of this range is an outlier, an observation that is distant from others

Uroflowmetry values also improved considerably, with mean values increasing of 3.26 ± 5.35 mL/s in the Group A and 5.6 ± 7.3 mL/s in the Group B. The difference between the two groups was again not statistically significant (*P* = 0.0638) (Fig. 2).

Uroflowmetry data reported in Table n°3 are also matched with voided volume and post voided residual (PVR) to assess the improvements from baseline. The percentage of patients reporting normal urine stream increased significantly, from 43.1 % at baseline to 54.5 % at visit 2 in Group A patients, and from 32.3 to 58.1 % in Group B patients.

**Fig. 3:**
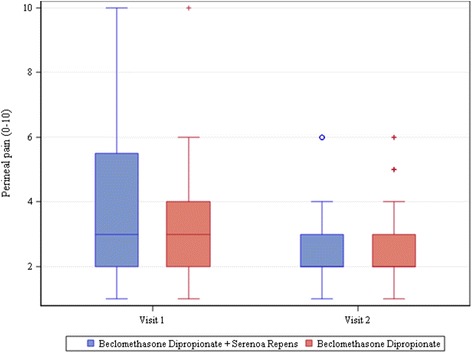
Perineal pain. The bottom of each box is the 25th percentile (Q1), the top is the 75th percentile (Q3), and the internal line is the median. The whiskers indicate variability outside the upper and lower quartiles, i.e. scores outside the middle 50 %. A circle outside of this range is an outlier, an observation that is distant from others

At visit 2, patients reported feeling less perineal pain, assessed by means of a Visual Analogue Scale of 0–10. The t-test for the difference between groups was not statistically significant although the p-value (0.0787) suggested a slightly stronger decrease among patients administered both BDP and SR (Fig. 3).

DREs were performed to evaluate prostate volume, temperature and consistency. A clear trend was not apparent for size; an enlarged prostate was detected in 50.5 % of the patients at visit 1, and 60.5 % at visit 2. On the contrary, the number of patients with a warm prostate decreased from 42.7 % of the patients at visit 1 to 5.5 % of the patients at visit 2. Clinical evidence of an inflamed prostate at DRE was reported in 83.8 % of the patients at visit 1, whereas 87.7 % of the patients presented a normal prostate at visit 2.

**Fig. 4:**
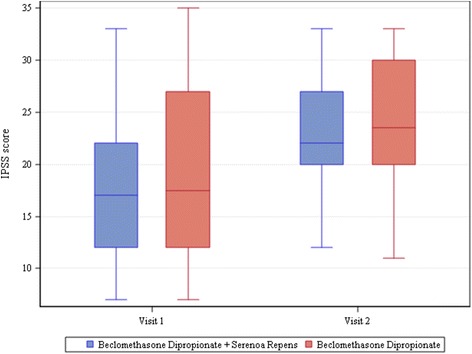
IPSS score. The bottom of each box is the 25th percentile (Q1), the top is the 75th percentile (Q3), and the internal line is the median. The whiskers indicate variability outside the upper and lower quartiles, i.e. scores outside the middle 50 %. A circle outside of this range is an outlier, an observation that is distant from others

The mean change from baseline in IPSS was 2.1 ± 7.9 (*P* = 0.0767) in Group A patients and 4.7 ± 7.9 (*P* <.0001) in Group B patients, which could suggest a worsening of IPSS score over time, although a temporary rise in score prior to final improvement is quite common in this pathology. The difference in the mean changes from baseline between the two groups was not statistically significant although a more marked increase of IPSS was present among those patients who took both treatments (Fig. 4).

PSA levels remained stable and below 4.0 ng/mL, dropping slightly from 3.4 ± 3.2 ng/mL (range 0.07–21 ng/mL) at visit 1 to 3.07 ± 2.35 at visit 2 (range 1.10–21.00 ng/mL).

Table 3 contains a summary of all the main efficacy results of the study, comparing the two groups of patients.

**Table 3:** Summary table

Variable	Treatment	
	Group A	Group B
	(*N* = 45)	(*N* = 136)
Age	53.3 ± 15.1	51.8 ± 14.9
IPSS		
Baseline value	21.6 ± 7.2	18.3 ± 6.4
Value at visit 2	23.7 ± 6.1	23.1 ± 5.7
Change from baseline	2.1 ± 7.9	4.7 ± 7.9
Test of change from baseline	*P* = 0.0767	*P* < .0001
Change vs baseline – test between two groups Two-Sample T-Test	*P* = 0.0593	
Voiding frequency (n/day)		
Baseline value	7.7 ± 2.5	7.8 ± 2.1
Value at visit 2	4.2 ± 1.4	4.1 ± 1.9
Change from baseline	−3.5 ± 2.7	−3.6 ± 2.8
Test of change from baseline	*P* < .0001	*P* < .0001
Change vs baseline – test between two groups Two-Sample T-Test	*P* = 0.8560	
Uroflowmetry (ml/s)		
Baseline value	13.1 ± 6.5	11.6 ± 3.8
Value at visit 2	16.4 ± 6.7	17.2 ± 6.5
Change from baseline	3.2 ± 5.3	5.6 ± 7.3
Test of change from baseline	*P* = 0.0002	*P* < .0001
Change vs baseline – test between two groups Two-Sample T-Test	*P* = 0.0638	
Voided volume and post voided residual PVR (ml)		
Baseline value	98.7 ± 20.5 45 ± 9.7	112.7 ± 19.3 51 ± 9.3
Value at visit 2	137.3 ± 33.9 32.4 ± 9.9	139.2 ± 25.3 30.1 ± 12.0
Change from baseline	38.6 ± 26.3 12.6 ± 9.1	26.5 ± 23.7 20.9 ± 11.4
Test of change from baseline	*P* = 0.0002	*P* < .0001
Change vs baseline – test between two groups Two-Sample T-Test	*P* = 0.0478	
Perineal pain		
Baseline value	3.2 ± 1.8	3.8 ± 2.0
Value at visit 2	2.5 ± 1.2	2.4 ± 1.1
Change from baseline	−0.6 ± 2.2	−1.3 ± 2.4
Test of change from baseline	*P* = 0.0699	*P* < .0001
Change vs baseline – test between two groups Two-Sample T-Test	*P* = 0.0787	
PSA		
Baseline value	2.8 ± 2.3	3.6 ± 3.5
Value visit 2	2.9 ± 2.1	3.1 ± 2.4
Change from baseline	0.1 ± 2.5	−0.5 ± 3.7
Test of change from baseline	0.5837	0.5036
Change vs baseline – test between two groups Two-Sample T-Test	*P* = 0.3613	

No adverse reactions or adverse events were reported.

## Discussion

Chronic inflammation plays an important role in the initiation and progression of a wide spectrum of diseases with prostate involvement [17]. Therefore, anti-inflammatory medications are commonly used in clinical practice for the treatment of several prostatic diseases, including nonbacterial prostatitis. These therapies aim principally at reducing symptoms caused by inflammation (e.g. pelvic pain, voiding dysfunction) that can significantly impair a patient’s quality of life [1, 5, 6].

The vast majority of our patients presenting with lower urinary tract inflammation were affected with nonbacterial prostatitis (85 %). This was an expected result given the high prevalence of this pathological condition [2, 6, 7]. In fact, nearly 50 % of all men experience prostatitis-like symptoms at least once during their lifetime and 90 % of those have abacterial prostatitis [2, 6, 7].

The majority of patients (152/180) underwent a 20-day course of therapy with BDP suppositories because of the severity of symptoms and the high compliance expected.

Treatment with Serenoa repens is very common and is driven by evidence-based practice to treat voiding and mainly storage symptoms of the lower urinary tract [18–20]. Its widespread use in clinical practice for the treatment of voiding symptoms is also described in several studies [21, 22]. Its beneficial effects are linked mainly to its pro-apoptotic and anti-proliferative properties, which are mediated by various mechanisms including inhibition of 5α-reductase, competition with dihydrotestosterone for binding to its receptor and inhibition of fibroblast-growth factor.

BDP suppositories were already found to be a safe and well-tolerated medication in previous studies [10, 11]. One-hundred percent treatment compliance and the absence of adverse reactions in our study substantiate its good safety profile also in inflammations of the lower urinary tract.

The vast majority of patients showed a clinically significant improvement of symptoms at visit 2. In fact, voiding parameters (frequency, uroflowmetry and urine stream) and perineal pain significantly improved during the study, likely with a positive effect on patients’ quality of life and perception of good clinical outcome.

We were not able to define a clear trend of improvement of the parameters evaluated by DRE because of the non-standardizable nature of the assessment. However, we did observe a tendency toward normalization in temperature and consistency of the prostate. As expected, PSA levels remained stable since it is not a specific parameter for lower urinary tract inflammation.

In our patients, IPSS increased at visit 2. This was however expected as it is a consequence of the way IPSS is intended to be used in common clinical practice [14]. The mean change from baseline was 2.1 ± 7.9 (*P* = 0.0767) in patients taking only BDP and 4.7 ± 7.9 (*P*<.0001) in patients also taking SR. This may indicate a worsening of IPSS, especially in patients taking both treatments, although a temporary increase in score prior to final improvement is quite common in inflammations of the lower urinary tract [23]. In fact, IPSS is more accurate for the evaluation of voiding symptoms, whereas BDP is an anti-inflammatory medication and is therefore meant to act mainly on symptoms of the lower urinary tract defined as storage symptoms [24]. Secondly, IPSS echoes the patient's symptoms in the last 4 weeks and does not reflect the 1-day status at visit 2. Therefore, a complete remission of the lower urinary tract inflammation at visit 2 may not be related to an evident improvement in IPSS. Moreover, despite the fact that IPSS is a validated questionnaire, it reflects the patient's feelings and is surely less objective than the other tests performed [25]. As a last comment, we prevented adding bias to this study by not prescribing any alpha-blockers; this choice was made based on the evidence that a lower urinary tract inflammatory disease has to be treated to improve the patients' symptoms [26]. Consequently, IPSS is a tool more suitable for evaluating the long-term outcome of a medical or surgical treatment rather than for the first control after a course of therapy with an anti-inflammatory medication [27].

Given that a significant number of patients took both BDP and SR, we decided to perform a post-hoc analysis in order to exclude any confounding results consequent to the association therapy; none of the comparisons between groups of all the parameters evaluated (voiding frequency, uroflowmetry, perineal pain, IPSS and PSA) were found to be statistically significant. These results confirm the positive effects of BDP suppositories in the treatment of lower urinary tract inflammation. As this is the very first study of its kind, the effectiveness of BDP in lower urinary tract inflammation should be confirmed in a randomized, double-blinded, prospective study.

## Conclusion

Beclomethasone dipropionate proved to be a safe and tolerable drug for treating lower urinary tract inflammations as no adverse events or adverse reactions were reported during the course of the study. All the main parameters (voiding frequency, uroflowmetry, urine stream, perineal pain) improved, except for an increase in IPSS. No significant differences were observed between patients treated with only beclomethasone dipropionate and those also treated with serenoa repens. Although randomized, controlled studies are required to substantiate these findings, our preliminary clinical observations support the use of beclomethasone dipropionate rectal suppositories in male patients affected by lower urinary tract inflammation.

## Abbreviations

BDP, Beclomethasone dipropionate; DRE, Digital rectal examination; IPSS, International Prostate Symptom Score; PSA, Prostate Specific Antigen; PVR, Post voided residual; SR, Serenoa repens.
